# Dietitians vary by counseling status in bean promotion with type 2 diabetes clients: A pilot study

**DOI:** 10.1002/fsn3.1578

**Published:** 2020-04-20

**Authors:** Donna M. Winham, Rebecca R. Nikl, Andrea M. Hutchins, Rose L. Martin, Christina G. Campbell

**Affiliations:** ^1^ Food Science & Human Nutrition Iowa State University Ames IA USA; ^2^ UnityPoint Health Des Moines Des Moines IA USA; ^3^ Human Physiology and Nutrition University of Colorado Colorado Springs Colorado Springs CO USA

**Keywords:** attitudes, dietetic practice, knowledge, legumes, pulses, type 2 diabetes mellitus

## Abstract

Beans are noted for their beneficial effects on blood glucose for persons with type 2 diabetes mellitus (T2DM). However, little is known about dietitian attitudes and perceptions, self‐efficacy, or counseling practices about beans in T2DM management. Through an online survey, the attitudes and perceptions dietitians have toward the role of beans in managing T2DM were examined. The practice intentions for advising T2DM clients about beans, perceived self‐efficacy for counseling on general nutrition topics and specifically on beans, were evaluated. While the target population was dietitians, all persons on the Arizona Dietetic Association and the Arizona School Nutrition Association listservs received a direct email invitation for an online survey on foods and chronic disease. There was no mention of beans or pulses to reduce bias toward bean advocates. Of the 302 dietitian respondents, over 66% counseled clients with T2DM. Fewer clinical counseling dietitians recommended beans to control blood glucose (*p* = .041) or to increase fiber (*p* < .05), and more of them promoted beans as being the same as other carbohydrates (*p* = .002). Higher mean self‐efficacy scores for general nutrition counseling were observed for T2DM counseling RDs (*p* < .001). Counseling dietitians in nonclinical settings had the highest bean self‐efficacy score (*p* < .001). Findings suggest clinical counseling dietitians are aware of bean health benefits, but do not consistently suggest beans to improve nutrition for those with T2DM in contrast to dietitians who counsel in other settings.

## Graphical abstract

Scientific evidence supports that eating beans is helpful in managing type 2 diabetes with adults. This study investigated Registered Dietitians' (RDs) views on beans and if they recommended to persons with type 2 diabetes. Findings suggest that RDs who do not counsel for diabetes in clinical settings are more likely to recommend them to clients.

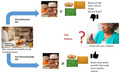

## 1 INTRODUCTION

The prevalence of type 2 diabetes mellitus (T2DM) has nearly doubled from 1980 to 2014, with an estimated 422 million adults with the condition globally(Roglic, 2016). In the United States (US), over 30 million people are living with T2DM, while an additional 84 million Americans have prediabetes—a precursor to the condition (Centers for Disease Control & Prevention, 2017). As the 7th leading cause of death in America, T2DM is a major contributor to cardiovascular disease, blindness, renal disease, and lower‐limb amputations (Centers for Disease Control & Prevention, 2017). The estimated annual cost for T2DM treatment is $245 billion in the United States (Centers for Disease Control & Prevention, 2017). In comparison, the total cost of treating all types of cancer is about $124.6 billion each year, or roughly half (Mariotto, Robin Yabroff, Shao, Feuer, & Brown, 2011). The most effective T2DM prevention and treatment are diet and lifestyle management, followed by pharmaceutical therapy (Diabetes Prevention Program Research Group, 2009).

Beans and other pulses such as peas, lentils, and chickpeas are naturally high in protein, fiber, folate, iron, phenolic compounds, and have a low‐glycemic index (Mitchell, Lawrence, Hartman, & Curran, 2009). Beans have gained recognition for their beneficial effects on human health. The 2015 Dietary Guidelines for Americans recommends 1.5 to 3 cup equivalents per week for males and 1 to 2 cup equivalents per week for females (US Department of Health & Human Services, 2017). The majority of pulses consumed in the United States are beans (*Phaseolus vulgaris* L.) (Bond, 2017). Regular pulse consumption has beneficial biological effects on the management of T2DM (Sievenpiper et al., 2009). In a meta‐analysis, when pulses, including beans, were given as part of a low‐glycemic index diet or a high‐fiber diet, clients had an absolute reduction in hemoglobin A1c (HbA1c) value of 0.48% (Sievenpiper et al., 2009). The US Federal Drug Administration proposed a threshold value decline of ≥0.3% in HbA1c as a biologically important change for clinical oral hypoglycemic agents (US Food & Drug Administration, 2008). Thus, a diet rich in beans or pulses would meet the FDA’s guideline for a meaningful change in HbA1c.

The physiological mechanism for this reduction in blood glucose levels when consuming a diet rich in pulses and beans is similar to that of acarbose, a common drug used in the management of T2DM. Acarbose functions by inhibiting the enzyme α‐amylase required to breakdown starch and thus may increase satiety. Both changes have been proven to aid in the management and control of T2DM (Barrett & Udani, 2011). Beans are high in phytates which have also been shown to inhibit α‐amylase as well as reduce calcium, a cofactor for activating α‐amylase. Bean extract has been tested and marketed for use in T2DM management (Barrett & Udani, 2011).

The diabetes prevention program (DPP, 2003) uses dietary compliance and exercise to prevent or slow individuals with prediabetes from progressing to T2DM. The dietary intervention is based on the US Food Pyramid and the National Cholesterol Education Program, both of which incorporate beans. Research with DPP participants found lifestyle changes alone reduced risk of developing T2DM by 58% (Diabetes Prevention Program Research Group, 2009). In contrast, metformin, a widely prescribed T2DM drug, lowers the risk of developing T2DM by 31% in study participants (Diabetes Prevention Program Research Group, 2009). When comparing the long‐term cost outcomes, lifestyle changes alone are more cost‐effective than metformin (Diabetes Prevention Program Research Group, 2003, 2009; Grundy, 1993). In the face of rising healthcare costs, adding beans to the diet could be a cost‐effective way to produce meaningful improvements in HbA1c values (Abdullah, Marinangeli, Jones, & Carlberg, 2017).

Lifestyle changes and dietary adherence can be difficult, especially if culturally appropriate strategies are not used. The prevalence of T2DM is greatest among indigenous populations and ethnic minorities in the United States (Centers for Disease Control & Prevention, 2017) Effective dietary strategies to manage T2DM should be considerate of traditional dietary patterns on the management of diabetes (Caban, Walker, Sanchez, & Mera, 2008; Fileti, 2011). In the case of minority groups like Hispanics where beans and other pulses are staple foods, dietary adherence may improve if dietitians are knowledgeable and comfortable in recommending culturally sensitive food options (McArdle, Greenfield, Avery, Adams, & Gill, 2017).

Few studies have considered dietitian knowledge of the benefits of bean consumption for those with T2DM or dietitian recommendations of beans to their clients. As carbohydrates are the principal nutrient of concern in diabetes management, exploration on quantity and quality of carbohydrate recommendations from dietitians is of interest. Dietitians may have conflicting priorities when counseling patients with T2DM on carbohydrate intake (Fileti, 2011). With limited time to explain concepts to a newly diagnosed person with T2DM, nutrition and behavior change recommendations may be more general instead of describing carbohydrate source variations that may not be practical in a single visit (Caban et al., 2008). Specific information on what dietitians cover with clients was not asked in this exploratory study, but is an essential follow‐up item. Canadian researchers found that 68% of dietitians surveyed reported that they “often” or “always” recommended pulses for those with T2DM (Desrochers & Brauer, 2001). However, no prior data on whether US dietitians recommend pulses with similar frequency were found. The current study objectives were to: (a) describe attitudes and perceptions dietitians have on the role of beans in managing T2DM; (b) determine their practice intentions for advising about beans to T2DM clients; and (c) evaluate perceived self‐efficacy for counseling on general nutrition and specifically on beans.

## 2 MATERIALS AND METHODS

The target population was dietitians in the state of Arizona, USA. With agency permission, direct email invitations were sent to the listserv rosters of the Arizona Dietetic Association and the School Nutrition Association of Arizona in September 2012. The listservs included nondietitian members and students, but the exact percentage was not provided. The survey invitation subject heading asked for opinions on functional foods and chronic disease. There was no mention of beans or pulses in the recruitment materials to reduce bias toward bean advocates. Reminders were emailed twice, approximately one week apart. Completion of the survey was considered informed consent. As an incentive, respondents who selected an external link and entered their mailing address received $3 in coupons for food products and/or could supply their email address for a raffle for one $50 gift card to Amazon.com randomly selected from every 50 completed surveys. The Arizona State University Institutional Review Board deemed the study exempt from further review due to minimal risk. Findings on dietitian knowledge of bean health benefits have been reported elsewhere (Winham, Hutchins, Thompson, & Dougherty, 2018).

The survey instrument was developed from a literature review, surveys on dietitian perceptions of functional foods, consumer surveys that explored similar topics, and formative interviews with five dietitians who worked in community nutrition, clinical nutrition, and diabetes education.(Cashman, Burns, & Otieno, 2003; Gobert & Duncan, 2009; Winham, Wooden, & Hutchins, 2014).

Demographic characteristics (age, gender, education, ethnicity, and race), employment status, workplace type, and dietary counseling experience questions were drawn from a previous survey of dietitian attitudes toward the preceptor role (Winham et al., 2014). Questions on personal bean consumption and knowledge of preparation were adapted from a validated food frequency questionnaire and similar consumer studies (Block, Gillespie, & Rosenbaum, 2000; Winham & Armstrong Florian, 2010).

Five Likert‐type statements on self‐efficacy in counseling were as follows: ability to provide specific nutrition information to patients, increase patient motivation, recommend specific dietary changes, give specific advice for maintaining dietary changes, and provide culturally sensitive counseling (Bandura, 2005). These same questions were asked at a later point in the survey specifically about self‐efficacy in counseling about beans. The response options for both questions were as follows: not confident at all, a little confident, somewhat confident, confident, or very confident.

Seven statements asked about the likelihood of giving bean advice to adults with T2DM. These included general bean nutrition characteristics (eat more beans for good nutrition, to increase your fiber, to help control blood glucose) and valid recommendations for T2DM (limit bean intake to one carbohydrate exchange per meal, limit bean intake to ½ cup or 15 g of carbohydrate per meal). Two questions addressed misconceptions (beans are the same as any other carbohydrate source, do not eat beans because you have T2DM). Likert‐type response categories were never or rarely, sometimes, often, almost always, or do not know. Noncounseling dietitians were instructed to answer these questions hypothetically.

Preliminary pilot testing with seven dietitians and 15 nutrition graduate students was used to refine the instrument. A second pilot test with 19 dietitians for feedback on construct and content validity was completed prior to official data collection.

Variables were examined for normality and distribution of responses. Based on the client type (clinical vs. community) and frequency distribution of reported percent time spent on counseling (none, more or less than 10% of time), the 302 dietitians were grouped into four categories. The first group was those who counseled adults with T2DM in clinical settings (40%, *n* = 121). The other categories were dietitians who counseled for T2DM in community or other locations (26%, *n* = 78), those who counseled for other conditions except T2DM in any practice type (11%, *n* = 33), and those who did not counsel at all (21%, *n* = 70). Self‐efficacy statements were summed to create a continuous score for general counseling (Cronbach's alpha 0.894), and bean counseling (Cronbach's alpha = 0.930). Differences in bean recommendations for adults with T2DM by counseling status were examined using Chi‐square or ANOVA. All reported statistical tests were two‐sided with significance set at *p* < .05 using IBM SPSS Statistics for Windows, version 24 (IBM Corp., Armonk, NY, USA).

## 3 RESULTS

**FIGURE 1:**
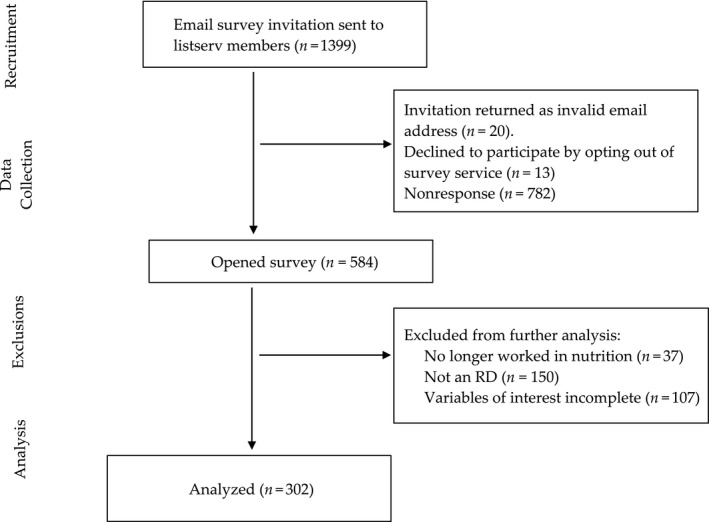
Consort flow diagram for participants of the Arizona dietitian type 2 diabetes client survey

The consort diagram in Figure 1 shows the flow of sampling contacts and respondents. Twenty of the 1,399 direct email invitations were returned as invalid addresses, and 13 individuals opted out of receiving further emails. Of the remaining contacts, 782 were nonresponsive and 584 began the survey (42%).

The majority of respondents were female (95.4%), white (92.7%), and non‐Hispanic (92.4%). Table 1 shows the demographics and bean consumption practices by counseling status. The dietitians who counseled but not for T2DM were significantly younger than their peers and had fewer years of experience (*p* < .001). About half of all respondents had a master's degree. The majority of respondents ate beans 1–2 times per week, reported knowing how to cook beans, and about 20% cooked beans 2–3 times per month. All of the 19 (6.3% of total) participants who indicated they were Certified Diabetes Educators counseled for T2DM (4 nonclinical; 15 clinical; data not shown).

**TABLE 1:** Demographic characteristics and bean consumption practices of dietitians by T2DM† counseling status (mean ± *SD*, or percentage) (*n* = 302)

Characteristics	Total	Do not counsel 23% (70)	Dietitians who counsel		
			Not T2DM† 11% (33)	T2DM nonclinical 26% (78)	T2DM clinical 40% (121)
	Mean ± standard deviation				
Age in years ***	43.8 ± 12.6	47.0 ± 11.5	37.2 ± 11.4	45.0 ± 12.8	43.0 ± 12.8
Years counseling experience***	11.0 ± 10.4	4.4 ± 7.0	9.5 ± 8.6	14.1 ± 10.1	13.3 ± 11.1
Percent time counseling past 2 years***	41 ± 35	0	44 ± 31	48 ± 34	59 ± 28
	%				
Education**					
Bachelors	43.5	36.8_a_	50.0_a,b_	31.2_a_	53.8_b_
Masters	49.7	51.5_a_	46.9_a_	57.1_a_	44.4_a_
Doctorate	6.8	11.8_a_	3.1_a,b_	11.7_a_	1.7_b_
Practice area***					
Clinical	44.6	10.8_a_	25.0_a_	0_b_	100_c_
Community	24.0	27.7_b_	46.9_a,b_	46.8_b_	0_c_
Food service management	12.2	33.8_b_	6.3_b_	14.3_b_	0_c_
Education and research	12.2	24.6_a_	12.5_a_	19.5_a_	0_b_
Consultation or private practice	7.0	3.1_a,b_	9.4_b,c_	19.5_c_	0_a_
Bean consumption frequency					
Once a month or less	6.9	6.0	6.3	9.2	6.1
2–3 times per month	22.1	29.9	15.6	15.8	23.7
1–2 times per week	48.1	50.7	46.9	46.1	48.2
3–4 times per week	17.6	9.0	31.3	19.7	17.5
5+ times per week	5.2	4.5	0	9.2	4.4
Dry bean cooking knowledge					
Knows how to cook beans	87.9	88.1	84.4	92.1	86.0
Bean cooking frequency					
Never	29.5	26.9	40.6	23.7	31.9
Once a month or less	44.1	44.8	37.5	48.7	42.5
2–3 times per month	19.1	20.9	18.8	17.1	19.5
1+ times per week	7.3	7.5	3.1	10.5	6.2

Note

Same subscript letters indicate column proportions that are not significantly different.

^†^ T2DM—type 2 diabetes mellitus.

** *p* < .01;

*** *p* < .001.

Table 2 shows the percentage distribution of responses to seven Likert‐type questions on bean recommendations for T2DM by dietitian counseling categories. Significantly fewer of the clinical counseling dietitians indicated they would recommend beans for good nutrition or to increase fiber to their T2DM clients in contrast to the other three groups. Clinical counseling dietitians were less likely to state that they would “often/always” suggest eating beans to “help control blood glucose.” Over 33% of clinical counseling dietitians agreed that they would “often/always” state that ‘beans were the same as any other carbohydrate source.” Only a few of all respondents would recommend adults with T2DM to “not eat beans” because of their condition.

**TABLE 2:** Frequency of recommendations about beans to persons with T2DM† by dietitian counseling categories (*n* = 302)

How frequently do you (or would you) make these recommendations to a person with T2DM about eating beans?	Total	Do not counsel 23% (70)	Dietitians who counsel		
			Not T2DM^†^ 11% (33)	T2DM nonclinical 26% (78)	T2DM clinical 40% (121)
	%				
1. Eat more beans for good nutrition*					
Never or rarely	2.3	1.4_a_	0_a_	1.3_a_	4.2_a_
Sometimes	16.3	8.7_a_	15.2_a,b_	16.7_a,b_	20.8_b_
Often/Almost always	81.3	89.9_a_	84.8_a,b_	82.1_a,b_	75.0_b_
2. Eat beans to increase your fiber*					
Never or rarely	0.7	0_a_	0_a_	0_a_	1.7_a_
Sometimes	7.0	4.3_a_	3.1_a_	3.8_a_	11.8_a_
Often/Almost always	92.3	95.7_a_	96.9_a,b_	96.2_a_	86.6_b_
3. Eat beans to help control blood glucose*					
Never or rarely	14.5	6.1_a_	6.1_a,b_	17.9_b_	19.2_b_
Sometimes	30.3	27.3_a_	33.3_a_	24.4_a_	35.0_a_
Often/Almost always	55.2	66.7_a_	60.6_a,b_	57.7_a,b_	45.8_b_
4. Limit beans to 1 carbohydrate exchange per meal					
Never or rarely	50.9	37.3	37.5	55.8	57.9
Sometimes	32.2	39.0	43.8	29.9	7.3
Often/Almost always	17.1	23.7	18.8	14.3	14.9
5. Limit bean intake to ½ cup or 15g of carbohydrate per meal					
Never or rarely	45.3	36.7	40.6	45.5	50.8
Sometimes	32.2	36.7	34.4	37.7	25.8
Often/Almost always	22.5	26.7	25.0	16.9	23.3
6. Beans are the same as any other carbohydrate source**					
Never or rarely	55.5	65.2_a_	60.6_a_	66.7_a_	41.3_b_
Sometimes	21.9	20.3_a_	27.3_a_	15.4_b_	25.6_a_
Often/Almost always	22.6	14.5_a_	12.1_a_	17.9_a_	33.1_b_
7. Do not eat beans because you have T2DM					
Never or rarely	98.0	98.0	100	98.7	96.7
Sometimes	1.0	0	0	1.3	1.7
Often/Almost always	1.0	1.5	0	0	1.7

Note

Same subscript letters indicate column proportions that are not significantly different.

^†^ T2DM—type 2 diabetes mellitus.

* *p* < .05;

** *p* < .01.

Table 3 shows the self‐efficacy reports by counseling cohorts on their perceived ability to guide patients on general nutrition‐related topics and specific bean‐related topics. Clinical counseling dietitians reported significantly greater confidence in their ability to counsel on almost all the individual general statements and had higher average values on the summary scale for general nutrition. The nonclinical counseling dietitians had similar responses to their clinical peers but with a significantly higher bean self‐efficacy scale.

**TABLE 3:** Self‐efficacy of dietitians for counseling clients on nutrition‐related topics and on beans by T2DM† counseling status (*n* = 302)

	Total	Do not counsel 23% (70)	Dietitians who counsel		
			Not T2DM† 11% (33)	T2DM nonclinical 26% (78)	T2DM clinical 40% (121)
	**%**				
How confident are you in your ability to counsel others on nutrition‐related topics?					
Provide specific nutrition information to clients***					
Not or a little confident	2.0	8.6_a_	0_a,b_	0_b_	0_b_
Somewhat confident	8.3	27.1_a_	6.1_b_	3.8_b_	0.8_b_
Confident	34.4	40.0_a,b_	57.6_b_	35.9_a,c_	24.0_c_
Very confident	55.3	24.3_a_	36.4_a_	60.3_b_	75.2_c_
Increase client motivation***					
Not or a little confident	4.3	15.7_a_	0_a_	1.3_b_	0.8_b_
Somewhat confident	22.2	35.7_a_	21.2_a,b_	17.9_b_	17.4_b_
Confident	42.4	27.1_a_	51.5_b_	43.6_b_	47.9_b_
Very confident	31.1	21.4_a_	27.3_a,b_	37.2_b_	33.9_a,b_
Recommend specific dietary changes***					
Not or a little confident	2.0	7.1_a_	0_a,b_	0_b_	0.8_b_
Somewhat confident	6.3	21.4_a_	6.1_b_	2.6_b,c_	0_c_
Confident	38.1	50.0_a_	51.5_a_	35.9_a,b_	28.9_b_
Very confident	53.6	21.4_a_	42.4_b_	61.5_c_	70.2_c_
Give specific advice for maintaining diet changes***					
Not or a little confident	3.0	11.4_a_	0_b_	0_b_	0.8_b_
Somewhat confident	9.3	21.4_a_	6.1_b_	6.4_b_	5.0_b_
Confident	41.4	45.7_a,b_	63.6_b_	35.9_a,c_	36.4_a_
Very confident	46.4	21.4_a_	30.3_a_	57.7_b_	57.9_b_
Provide culturally sensitive counseling*					
Not or a little confident	9.9	20.0_a_	9.1_a,b_	7.7_b_	5.8_b_
Somewhat confident	30.5	40.0_a_	30.3_a_	28.2_a_	26.4_a_
Confident	38.7	30.0_a_	45.5_a_	39.7_a_	41.3_a_
Very confident	20.9	10.0_a_	15.2_a,b_	24.4_b_	26.4_b_
How confident are you in your ability to counsel others on bean nutrition‐related topics? (*n* = 288)					
Provide specific nutrition information about beans***					
Not or a little confident	10.1	22.4_a_	9.4_a,b_	3.9_b_	7.1_b_
Somewhat confident	25.0	38.8_a_	18.8_b_	21.1_b_	21.2_b_
Confident	41.6	26.9_a_	56.3_b_	56.6_b_	51.3_b_
Very confident	17.4	11.9_a_	15.6_a_	18.4_a_	20.4_a_
Increase client motivation to eat beans*					
Not or a little confident	9.4	19.4_a_	6.3_a,b_	2.7_b_	8.9_b_
Somewhat confident	26.6	35.8_a_	18.8_a_	25.3_a_	24.1_a_
Confident	46.9	34.3_a_	56.3_b_	53.3_b_	47.3_b_
Very confident	17.1	10.4_a_	18.8_a_	18.7_a_	19.6_a_
Recommend specific dietary changes to include beans***					
Somewhat confident	17.4	31.3_a_	9.4_b_	16.0_b_	12.4_b_
Confident	49.5	37.3_a_	68.8_b_	52.0_a,b_	49.6_a,b_
Very confident	23.7	11.9_a_	18.8_a,b_	30.7_b_	27.4_b_
Give specific advice to maintain bean consumption**					
Not or a little confident	12.2	11.4_a_	0_b_	0_b_	0.8_b_
Somewhat confident	23.4	21.4_a_	6.1_b_	6.4_b_	5.0_b_
Confident	45.1	45.7_a,b_	63.6_b_	35.9_a,c_	36.4_a_
Very confident	19.2	21.4_a_	30.3_a_	57.7_b_	57.9_b_
Provide culturally sensitive counseling about beans**					
Not or a little confident	23.7	37.3_a_	21.9_a,b_	14.7_b_	22.1_b_
Somewhat confident	32.4	34.3_a,b_	28.1_a,b_	42.7_b_	25.7_a_
Confident	30.0	19.4_a_	43.8_b_	26.7_a,b_	34.5_b_
Very confident	13.9	9.0_a_	6.3_a_	16.0_a_	17.7_a_
Counseling self‐efficacy score***	20.9 ± 3.3	18.3 ± 4.0	20.6 ± 2.7	21.6 ± 2.6	21.9 ± 2.7
Bean counseling self‐efficacy score***	18.2 ± 4.3	16.1 ± 4.8	18.8 ± 3.5	19.2 ± 3.4	18.6 ± 4.2

Note

Same subscript letters indicate column proportions that are not significantly different from each other.

^†^ T2DM—type 2 diabetes mellitus.

* *p* < .05

** *p* < .01

*** *p* < .001.

## 4 DISCUSSION

Dietitians are highly influential in the nutritional choices of adults with T2DM and other health conditions. Strong evidence supports the effectiveness of nutrition interventions and counseling provided by RDs (Early & Stanley, 2018; Sialvera et al., 2018). However, effectiveness is contingent on the quality and accuracy of the information recommended. The results of our study showed positive attitudes toward beans in general by RDs, but differences in practices and self‐efficacy toward recommending beans to adults with T2DM based on dietitian counseling or noncounseling status. Arizona dietitians who counseled in clinical settings were less likely to encourage T2DM clients to consume beans than those who counseled in nonclinical settings. Despite lower bean consumption recommendations, dietitians who counsel for T2DM reported a much higher self‐efficacy in ability to counsel on general nutrition topics than their peers. Diabetes Specialist Dietitians in the United Kingdom were also found more confident in their abilities to counsel on quantity of carbohydrates and more likely to counsel on carbohydrate restriction than nondiabetes specialist dietitians (Sialvera et al., 2018). Parker et al. found South African health professionals had high personal views of their self‐efficacy but were overconfident in their knowledge when queried on factual information (Parker, Steyn, Levitt, & Lombard, 2011). Hand and Abram identified similar concerns of self‐confidence as a barrier to willingness to practice in the face of new evidence in the dietetics profession (Hand & Abram, 2016).

A Canadian dietitian survey suggests that beans may not be consistently recommended to individuals with T2DM there as well. Canadian dietitians were asked how frequently they recommend legumes, a broader category term that includes pulses like beans, for different health conditions during counseling. Eighty‐seven percent stated they recommend legumes to clients with cardiovascular disease, but only 68% reported that they recommend legumes to individuals with T2DM (Desrochers & Brauer, 2001). While most Canadian dietitians did recommend legumes to T2DM clients, it is concerning that there was a discrepancy between these two chronic diseases at all. It is possible that dietitians may be more aware of the benefits of bean consumption on blood lipids and less familiar with their effect on postprandial blood glucose (McArdle et al., 2017; Sialvera et al., 2018; Winham et al., 2018).

These practice behaviors directly affect ethnic groups who traditionally consume beans as staple foods. After a T2DM diagnosis, Hispanics often report suffering physically and emotionally without adequate knowledge of their condition.(Hu, Amirehsani, Wallace, & Letvak, 2013) These individuals often feel they must give up traditional foods, including beans, and family events involving food, which results in a sense of loss and conflict.(Caban et al., 2008) Dietitians and other health professionals who work with these populations need to give more guidance and education that includes the importance and benefits of bean consumption (Caban et al., 2008; Early & Stanley, 2018; Fileti, 2011).

Diffusion of new therapies in biomedical fields is an inherently slow process (Rodgers, 2003). On average, an innovative treatment takes 17 years to reach patients in an academic medical center after efficacy testing. Practice change takes even longer to reach community‐based settings.(Hand & Abram, 2016) A barrier to the acceptance of a new therapy is the concern that it does not apply to a specific population of clients.(Hand & Abram, 2016; Manore et al., 2017).

A strategy that may advance acceptance and distribution of pulse recommendations involves the engagement of opinion leaders such as the Dietary Guidelines for Americans (DGA), Academy of Nutrition and Dietetics, and the American Diabetes Association. Both the 2015 DGA and Academy of Nutrition and Dietetics advise the public or dietetics professionals to increase intake of beans because of their nutritional value.(Academy of Nutrition & Dietetics Evidence Analysis Library, 2015; US Department of Health & Human Services, 2017) However, the messages regarding bean intake are vague. In disease‐specific information on healthy eating for T2DM, the Academy of Nutrition and Dietetics recommends food choices high in fiber, and lower in fat and sodium, yet there is no specific mention of beans, though they contain all these attributes (Academy of Nutrition & Dietetics Evidence Analysis Library, 2015). The 2015 DGA recommends intake of beans (legumes) several times a week for their nutrient density and higher fiber content. The DGA does highlight the bioactive components in legumes which could improve vascular function and the lipid‐lowering effects of soluble fiber (Flock & Kris‐Etherton, 2011; US Department of Health & Human Services, 2017) This is one of the most positive and specific messages regarding bean intake.

The American Diabetes Association recommends including beans as part of a healthy diet. Beans and legumes are touted as having a low‐glycemic index for those using it to plan meals, but the two popular methods for meal planning do not make any special mention of beans as being different than other starchy foods. If using the “plate method,” only ¼ of the plate is recommended to come from starchy foods. For those who use carbohydrate counting, legumes are lumped into the starchy food category as well. The protein content of beans is not addressed, nor is mention made of beans as a meat substitute (American Diabetes Association, 2017). The fiber recommendations of the American Diabetes Association are no greater than that of the standard Dietary Reference Intake, as it is thought individuals with T2DM would find it difficult to sustain a fiber intake greater than the average of less than 24 g/day (American Diabetes Association, 2017).

Study limitations include the use of a convenience sample drawn only from dietitians in Arizona. Respondents were not asked why or how they make decisions about dietary recommendations to clients with T2DM. RDs were not asked about specifics of the client counseling interaction. Time constraints may limit the ability of clinical RDs to discuss inclusion of beans in the diet. Future studies should gather information about the nature of counseling sessions to clarify this aspect. The survey questions focused on beans only and did not inquire about other pulses such as peas and lentils. These results may not be applicable to other Arizona dietitians, or dietitians overall. The self‐administered instrument may have contained written questions that respondents did not understand, even though efforts were made to pilot test the survey for comprehension among dietitians.

## 5 CONCLUSIONS

The current research is one of a few investigations on dietitian recommendations or advice about beans to adults with T2DM. Dietitians who counsel individuals with T2DM may benefit from additional education on the health benefits of beans and other pulses. There are increased interests in the use of functional foods such as beans as an alternative to pharmaceutical medications (Sikand, Kris‐Etherton, & Boulos, 2015). Additional studies should be conducted to determine why there is a difference between clinical and nonclinical dietitians regarding bean recommendations to those with T2DM. Understanding the reasons for this gap in bean recommendations between the cohorts of dietitians will help identify strategies to inform and address best practices.

## CONFLICTS OF INTEREST

The authors declare that they have no conflict of interest.

## ETHICAL STATEMENT

The study was conducted in accordance with the Declaration of Helsinki and was deemed exempt by the Iowa State University Institutional Review Board (#17‐301).
